# Clinical significance and related factors of rectal hyposensitivity in patients with functional defecation disorder

**DOI:** 10.3389/fmed.2023.1119617

**Published:** 2023-02-21

**Authors:** Ya Jiang, Yan Wang, Meifeng Wang, Lin Lin, Yurong Tang

**Affiliations:** Department of Gastroenterology, The First Affiliated Hospital with Nanjing Medical University, Nanjing, Jiangsu, China

**Keywords:** functional defecatory disorder, rectal hyposensitivity, Bristol stool formation scale, age, male

## Abstract

**Background:**

Rectal hyposensitivity (RH) is not uncommon in patients with functional defecation disorder (FDD). FDD patients with RH are usually unsatisfied with their treatment.

**Aims:**

The aim of this study was to find the significance of RH in patients with FDD and the related factors of RH.

**Methods:**

Patients with FDD first completed clinical questionnaires regarding constipation symptoms, mental state, and quality of life. Then anorectal physiologic tests (anorectal manometry and balloon expulsion test) were performed. Rectal sensory testing (assessing rectal response to balloon distension using anorectal manometry) was applied to obtain three sensory thresholds. Patients were separated into three groups (non-RH, borderline RH, and RH) based on the London Classification. The associations between RH and clinical symptoms, mental state, quality of life, and rectal/anal motility were investigated.

**Results:**

Of 331 included patients with FDD, 87 patients (26.3%) had at least one abnormally elevated rectal sensory threshold and 50 patients (15.1%) were diagnosed with RH. Patients with RH were older and mostly men. Defecation symptoms were more severe (*p* = 0.013), and hard stool (*p* < 0.001) and manual maneuver (*p* = 0.003) were more frequently seen in the RH group. No difference in rectal/anal pressure was found among the three groups. Elevated defecatory desire volume (DDV) existed in all patients with RH. With the number of elevated sensory thresholds increasing, defecation symptoms got more severe (r = 0.35, *p* = 0.001). Gender (male) (6.78 [3.07–15.00], *p* < 0.001) and hard stool (5.92 [2.28–15.33], *p* < 0.001) were main related factors of RH.

**Conclusion:**

Rectal hyposensitivity plays an important role in the occurrence of FDD and is associated with defecation symptom severity. Older male FDD patients with hard stool are prone to suffer from RH and need more care.

## Introduction

Approximately 50% of patients with functional constipation have difficulty in defecating (1) and may have the functional defecatory disorder (FDD) (2). FDD significantly affects productivity, mental health, and quality of life (QOL) (3).

Intact rectal sensation and motility are critical to normal bowel movement and defecation. The presence of sufficient stool and intact sensation will trigger the perception of rectal fullness through rectal afferent pathways (4). Rectal hyposensitivity (RH) refers to a blunted sensation of mechanical distension, which is indicated by the elevation of sensory thresholds beyond the normal range (5). As sensation and motility are inextricably linked, alteration in one domain can affect the other. RH, rectal motor dysfunction, and altered recto-anal reflex activity are particularly associated with FDD (6).

Patients with RH commonly present with constipation (48%) (7), and about 18%–68% of constipated patients have RH (8). It is reported that RH is more common in patients with functional disorders (i.e., dyssynergic defecation) rather than structural diseases (i.e., rectocele and intussusception) (9). Our team has found that RH is associated with defecation symptoms and specifies an eventual diagnosis of FDD over delayed gut transit (10).

Rectal hyposensitivity is associated with constipation, but its clinical importance remains unclear. In addition, little is known about the characteristics of FDD patients with RH and the related factors of RH in these individuals. Given the above deficiencies, we carried out this study to explore the influence of RH on constipation symptoms, mental state as well as QOL, and related factors of RH in an FDD population.

## Methods

### Participants

This is a cross-sectional study. We enrolled patients with FDD (Rome IV core criteria defined) who were referred to our gastrointestinal motility clinic between January 2014 and May 2021. Patients with pregnancy, drug-induced constipation, secondary constipation due to other diseases, a history of the prior bowl or anorectal surgery, or an abuse history were excluded. The study protocol was approved by the Ethics Committee of the First Affiliated Hospital with Nanjing Medical University (2022-SR-210).

All of our target patients underwent high-resolution anorectal manometry (HR-ARM) and balloon expulsion test (BET) and completed the required questionnaires.

### High-resolution anorectal manometry

A high-resolution solid-state anorectal manometry device (Manoscan AR 360; Given Imaging, Yokneam, Israel) with 12 sensors was adopted to evaluate patients’ defecation function. The absolute parameters were assessed as follows: anal resting pressure (20–30 s), anal sphincter length, duration of the sustained squeeze, anal pressure during squeeze (three attempts for a maximum duration of 20–30 s), rectal pressure, and anal residual pressure during attempted defecation (typically 20–30 s, three times, with a 2-min rest interval). Comprehensive parameters were also collected for analysis including manometric defecation index (MDI), recto-anal pressure gradient (RAG), and anal relaxation rate during attempted defecation (11).

The rectal sensation was evaluated by incrementally distending the rectal balloon by 10 mL from 0 to 400 mL, and the thresholds for first constant sensation volume (FCSV), defecatory desire volume (DDV), and maximum tolerable volume (MTV) were recorded.

### Upper limits of normal rectal sensation (95%)

A previously published dataset of 54 healthy individuals (35 women) assessed by our motility center (Table 1) was used to define the upper limits of normal (95%) for three sensory thresholds (men and women have different upper limits of normal) (10). The healthy individuals did not have any surgical history related to constipation and they all had normal bowel movements.

**Table 1:** Rectal sensory thresholds (mLs) in 54 healthy individuals (35 women) by gender.

Rectal sensory thresholds [upper limits of normal (95.0%)]	Females (*n* = 35)	Males (*n* = 19)
FCSV (mL)	90	70
DDV (mL)	170	120
MTV (mL)	320	250

FCSV: first constant sensation volume; DDV: defecatory desire volume; MTV: maximum tolerable volume.

### Diagnostic criteria for RH

According to the London Classification published in Jan 2020, RH is defined as an abnormal elevation of ≥2 sensory thresholds while borderline RH refers to one of the three sensory thresholds exceeding the upper limit of the normal range (12).

### Balloon expulsion test

A 4-cm long balloon filled with 50 mL of warm water was placed in the patient’s rectum while the patient was seated on a commode and was asked to expel the balloon, in privacy. If the subject could not expel the balloon after 1 min of straining, it was deflated and removed and the result was identified as abnormal (13).

### Defecography

Patients who were suspected to suffer from rectal structural diseases such as rectocele or intussusception underwent defecography. The presence of poor opening of the anorectal angle, poor relaxation of the anal canal, or poor expulsive effort generated which is related to retention of more than 50% contrast was defined as abnormal (14).

### Questionnaires

#### Constipation symptoms

Patients with FDD were asked about their spontaneous bowel movements (SBMs) (times per week), defecation duration, and stool consistency evaluated by Bristol Stool Formation Scale (BSFS). In addition, Rome IV core criteria for functional constipation were adopted to evaluate symptoms including fewer bowel movements (<3 times per week), straining, feeling incomplete defecation, anal blockage, lumpy or hard stool, and manual maneuvers during the last 6 months (15). In addition to collecting the typical symptoms, we used Patient Assessment of Constipation Symptoms (PAC-SYM) (16) to measure patients’ subjective feelings about constipation, with higher scores indicating more severe symptoms.

#### Mental health

General Anxiety Disorder 7-item (GAD-7) (17) and Patient Health Questionnaire-9 (PHQ-9) (18) were adopted to measure anxiety and depression symptoms, respectively. Higher scores suggested more severe symptoms and a score of >5 indicated anxiety or depression state.

#### Quality of life

The Patient Assessment of Constipation Quality of Life (PAC-QOL) questionnaire specifically assesses constipated patients’ QOL (19). It contains 28 items divided into four subscales (physical discomfort, psychosocial discomfort, worry/anxiety, and satisfaction with treatment). Higher scores showed poorer constipation-related QOL.

### Statistical analysis

Statistical analyses were conducted with SPSS version 26.0. Continuous variables were presented as the mean ± SD or median (interquartile range). Categorical variables were given as relative frequencies. A one-way ANOVA test was used to compare normally distributed variables while a rank-sum test was used to compare non-normally distributed variables. Fisher’s exact test was adopted to analyze categorical variables. The Spearman correlation analysis was applied to find associations between clinical manifestations and three rectal sensory thresholds. And logistic regression was applied to explore related factors of RH in patients with FDD. *p*-values were corrected for multiple tests with the Bonferroni procedure. *p*-values less than 0.05 were considered statistically significant.

## Results

### Demographics

We enrolled 331 patients with FDD in total, of which 87 (26.3%) had at least one abnormally elevated rectal sensory threshold and 50 (15.1%) had two or three thresholds above the 95% normal upper limit. According to the latest published London Classification of anorectal function, these patients were divided into three groups (non-RH: n = 244 [73.7%]; borderline RH: n = 37 [11.2%]; and RH: n = 50 [15.1%]). Patients in the three groups were similar in BMI and constipation duration. Patients in RH and borderline RH groups were significantly older than those in the non-RH group (*p* = 0.005 and *p* = 0.036, respectively). In addition, more male patients were found in the RH group (male/female: 38/12, *p* < 0.001) compared to those in the non-RH group and borderline RH group. Detailed data are listed in Table 2.

**Table 2:** Demographic characteristics of patients stratified by rectal sensation in 311 patients with FDD.

Demographics	Non-RH (*n* = 244)	Borderline RH (*n* = 37)	RH (*n* = 50)	*p*
Age(yr), mean ± SD^a^	47.54 ± 16.78	53.73 ± 15.73	54.82 ± 16.81	0.005
Gender, male/female(%)^b^	81/163 (33.2)	15/22 (40.5)	38/12 (76.0)	<0.001
BMI(kg/m^2^), median (interquartile range)	21.97 (3.77)	21.88 (5.37)	22.86 (3.73)	0.212
Constipation Duration(yr), median (interquartile range)	6.00 (9.50)	3.00 (5.75)	5.00 (6.63)	0.148

BMI: body mass index; yr: year.

a The patients in RH and Borderline RH groups were significantly older than those in Non-RH group (post hoc *p* = 0.005 and *p* = 0.036, respectively).

b More males were observed in RH group than those in Non-RH and Borderline-RH group (*v* value is defined to be a “discovery” using a Bonferroni procedure for multiple tests which controls the false discovery rate at 0.05).

### Functional tests

Analysis of grouped data suggested that all three rectal sensory thresholds were significantly high in the RH group (*P*s < 0.001) but no difference was observed between borderline RH and RH groups (Table 3). Patients with RH showed the lowest anal relaxation rate (*p* = 0.017), especially lower than those in the non-RH group (*p* = 0.013). However, the parameters regarding anorectal pressure and pelvic coordination did not differ among the three groups (all *P*s > 0.05), which could be referred to in Table 3. As regards to BET, FDD patients with more abnormally elevated sensory thresholds were more likely to fail it, but no significant difference was seen (*p* = 0.073).

**Table 3:** Comparisons of rectal/anal pressure, pelvic coordination and rectal sensory thresholds of patients stratified by rectal sensation in 311 patients with FDD.

HARM metrics	Non-RH (*n* = 244)	Borderline RH (*n* = 37)	RH (*n* = 50)	*p*
Anal resting pressure (mm Hg), mean ± SD	88.67 ± 24.46	89.55 ± 20.86	85.97 ± 20.79	0.670
Maximum squeeze pressure(mm Hg), median (interquartile range)	218.35 (92.65)	227.00 (99.15)	241.05 (149.53)	0.405
Duration of sustained squeeze (s), median (interquartile range)	19.25 (9.00)	19.80 (7.80)	15.30 (14.72)	0.296
Rectal defecation pressure (mm Hg), median (interquartile range)	36.15 (23.28)	38.70 (24.35)	37.00 (42.15)	0.894
Anal residual pressure (mm Hg), median (interquartile range)	93.40 (44.67)	94.80 (52.40)	96.55 (42.55)	0.190
Anal relaxation rate(%), median (interquartile range)^a^	−4.05 (42.49)	−4.74 (47.00)	−19.58 (50.86)	0.017
RAG (mm Hg), mean ± SD	−55.14 ± 34.91	−59.30 ± 28.77	−62.16 ± 37.98	0.382
MDI, median (interquartile range)	0.40 (0.29)	0.36 (0.19)	0.38 (0.34)	0.566
Abnormal BET, *n* (%)	231 (94.67)	36 (97.30)	50 (100.00)	0.073
FCSV (mL), median (interquartile range)^b^	40.00 (20.00)	50.00 (50.00)	90.00 (70.00)	<0.001
DDV (mL), median (interquartile range)^b^	90.00 (40.00)	150.00 (115.00)	200.00 (115.00)	<0.001
MTV (mL), median (interquartile range)^b^	130.00 (70.00)	230.00 (60.00)	350.00 (170.00)	<0.001

RAG: recto-anal pressure gradient; MDI: manometric defecation index; BET: bollon expulsion test; FCSV: first constant sensation volume; DDV: defecatory desire volume; MTV: maximum tolerable volume.

a Anal relaxation rate of patients in RH group was significantly lower than that in Non-RH group(*p* = 0.013).

b FCSV, DDV and MTV of patients were higher in Borderline RH and RH groups compared to those in Non-RH group (*p* < 0.001).

^a b^*p* value is defined to be a “discovery” using a Bonferroni procedure for multiple tests which controls the false discovery rate at 0.05.

As shown in Table 4, FDD patients with RH were more likely to suffer abnormally elevated FCSV (*p* = 0.008), DDV, and MTV compared to patients with borderline RH, especially in DDV and MTV (both *P*s < 0.001), which indicated that most patients with FDD tended to have abnormally elevated DDV (45.9% in borderline RH group and 100% in RH group) rather than FCSV and MTV. In the RH group, nearly one-third (n = 16, 32%) of patients with FDD had three elevated rectal sensory thresholds.

**Table 4:** Comparison of the occurrence of each abnormally elevated rectal sensory threshold between Borderline RH and RH groups.

Abnormally elevated threshold	Borderline RH (*n* = 37)	RH (*n* = 50)	*p*
FCSV, *n* (%)	13 (35.1)	32 (64.0)	0.008
DDV, *n* (%)	17 (45.9)	50 (100)	<0.001
MTV, *n* (%)	7 (18.9)	34 (68)	<0.001

FCSV: first constant sensation volume; DDV: defecatory desire volume; MTV: maximum tolerable volume.

There were only weak links between FCSV and anal resting pressure (r = −0.155, *p* = 0.005), maximum squeeze pressure (r = −0.109, *p* = 0.047), as well as anal residual pressure (r = −0.148, *p* = 0.007). No other links between rectal sensory thresholds and motility parameters were found.

### Clinical manifestations

Higher score for defecation symptoms (including straining, incomplete or failed defecation, and low stool weights) in PAC-SYM (*p* = 0.013), lower score for BSFS (*p* = 0.019), greater proportion of assistance for defecation (*p* = 0.003), and higher presence of hard stool (*p* < 0.001) were reported by patients with RH (Table 5). However, no difference in GAD-7, PHQ-9, or PAC-QOL scores was shown in the three groups (all *P*s > 0.05). A weak correlation was found between defecation symptoms and mental state (GAD-7: r = 0.329, *p* = 0.002; PHQ-9: r = 0.371, *p* < 0.001). In addition, the score for defecation symptom was moderately related to PAC-QOL score (r = 0.570, *p* < 0.001) as well as scores for sub-scales in PAC-QOL (Physical Discomfort: r = 0.434, *p* < 0.001; Psychosocial Discomfort: r = 0.50, *p* < 0.001; and Worry/Anxiety: r = 0.499, *p* < 0.001).

**Table 5:** Constipation symptoms and defecation characteristics of 311 patients with FDD in 3 groups.

Constipation symptoms	Non-RH (*n* = 244)	Borderline RH (*n* = 37)	RH (*n* = 50)	*p*
SBMs (times per week), median (interquartile range)	2.0 (4.0)	3.0 (4.0)	2.0 (3.0)	0.638
BSFS, median (interquartile range)^a^	2.0 (3.0)	2.0 (3.0)	1.5 (1.0)	0.019
Defecation duration, median (interquartile range)	3.0 (1.0)	3.0 (1.0)	2.0 (2.0)	0.114
<3 defecations/week, *n* (%)	140 (57.4)	17 (45.9)	30 (60)	0.367
Hard stool, *n* (%)^b^	135 (55.3)	22 (59.5)	44 (88)	<0.001
Manual maneuvers, *n* (%)	65 (26.6)	2 (5.4)	19 (38)	0.003
Straining, *n* (%)	123 (50.4)	22 (59.5)	22 (44.0)	0.362
Feeling incomplete evacuation, *n* (%)	147 (60.2)	20 (54.1)	38 (76.0)	0.065
Feeling anal obstruction, *n* (%)	78 (32.0)	5 (13.5)	16 (32)	0.069
PAC-SYM Score				
Abdominal symptoms, median (interquartile range)	1.00 (1.00)	1.00 (1.38)	0.50 (1.50)	0.523
Rectal symptoms, median (interquartile range)	0.33 (1.00)	0.33 (0.67)	0.33 (1.00)	0.277
Defecation symptoms, median (interquartile range)^c^	2.40 (1.20)	2.20 (1.20)	2.80 (0.85)	0.013
Total score, median (interquartile range)	1.50 (0.92)	1.33 (0.42)	1.42 (0.68)	0.097

SBMs: spontaneous bowel movements; BSFS: Bristol stool formation scale.

a The score for BSFS in RH group was significantly higher than that in Non-RH group (*p* = 0.016).

b More patients suffered hard stools in RH group than those in Non-RH and Borderline-RH group.

c The score for Defecation Symptoms in RH group was significantly higher than that in Borderline RH group (*p* = 0.010).

^a b c^*p* value is defined to be a “discovery” using a Bonferroni procedure for multiple tests which controls the false discovery rate at 0.05.

### Related factors of RH and rectal sensory thresholds

**Figure 1:**
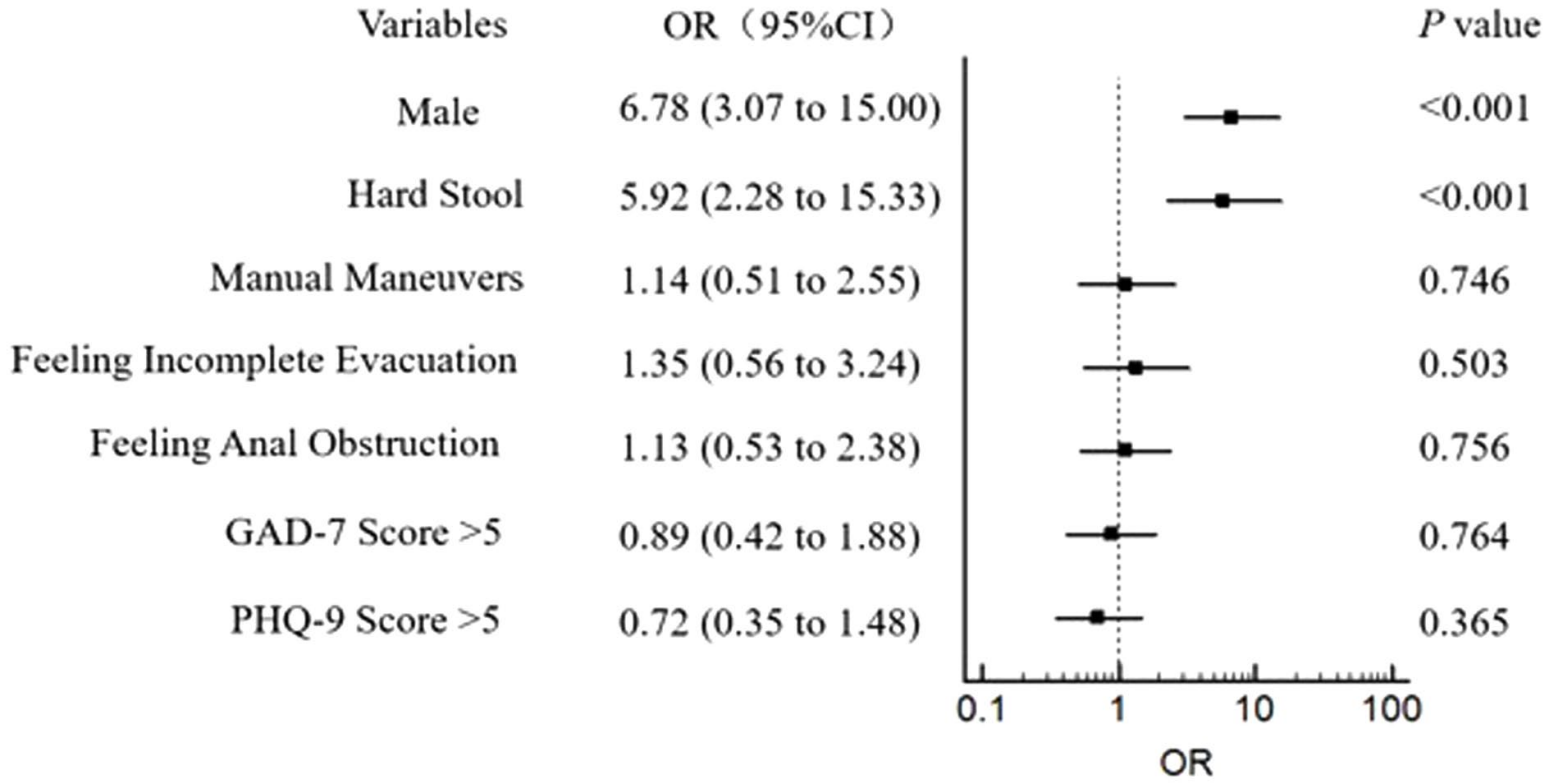
Assocaitions between RH and gender, symptoms of constipation, and mental state in 331 patients of FDD. RH: Rectal hyposensitivity; FDD: Functional defecation disorder; GAD-7: General anxiety disorder 7-item; PHQ-9: Patient health questionnaire-9. The outcome was adjusted for the potential confounding factors: age (years), BMI, and constipation duration.

According to our findings that some variables (age, gender, hard stool, manual maneuvers, feeling incomplete evacuation, feeling anal obstruction) made statistically significant changes at the 10% level and anxiety/depression could also interact with rectal sensation (20), we included them in the logistic regression model. Logistic regression revealed that gender (male) and hard stool were closely related to the occurrence of RH (Figure 1). Furthermore, spearman correlation analysis of clinical manifestations and rectal sensory thresholds suggested that FDD patients with older age and lower BSFS score (indicating hard/lumpy stool) were more likely to suffer abnormally elevated FCSV and DDV. In addition, older patients with higher PHQ-9 scores were prone to have abnormally elevated MTV. No correlation was observed among the PAC-SYM score, SBMs, GAD-7 score, and three rectal sensory thresholds (Table 6). An increasing number of abnormally elevated thresholds suggested a linear relationship with more severe defecation symptoms in PAC-SYM (r = 0.35, *p* = 0.001).

**Table 6:** Relationship between clinical manifestations and 3 rectal sensory thresholds of 311 patients with FDD.

	FCSV (mL)		DDV (mL)		MTV (mL)	
	*r*	*p*	*r*	*p*	*r*	*p*
Age	0.121	0.028	0.152	0.005	0.130	0.018
SBMs	−0.053	0.333	−0.050	0.369	−0.050	0.360
BSFS	−0.176	0.001	−0.116	0.036	−0.085	0.123
Abdominal symptoms	0.028	0.606	−0.032	0.564	−0.041	0.459
Rectal symptoms	0.002	0.972	0.049	0.374	−0.043	0.436
Defecation symptoms	0.079	0.153	0.086	0.116	0.073	0.185
PAC-SYM score	0.032	0.561	0.015	0.779	−0.002	0.970
GAD-7	0.040	0.469	0.023	0.681	0.053	0.334
PHQ-9	0.019	0.737	0.055	0.320	0.122	0.026

SBMs: spontaneous bowel movements; BSFS: Bristol stool formation scale; FCSV: first constant sensation volume; DDV: defecatory desire volume; MTV: maximum tolerable volume; PAC-SYM: patient assessment of constipation symptoms; GAD-7: general anxiety disorder 7-item; PHQ-9: patient health questionnaire-9.

## Discussion

Rectal hyposensitivity was reported in almost 25% of adult patients with chronic idiopathic constipation (21, 22). RH was associated strongly with pelvic floor dysfunction other than abnormal motility. A recently published study revealed that patients with three abnormally elevated sensory thresholds suffered almost two times as frequent defecation disorder as patients with normal rectal sensation (44.3% vs. 23.2%) (23). However, in a study that enrolled 107 patients with FC (37.4% had RH), no significant difference in RH was observed between the non-FDD and FDD groups (24). The impaired rectal sensation may be negatively associated with abnormal rectal/anal pressure and paradoxical pelvic contraction. Biofeedback therapy (BFT) is the first-line treatment for FDD but patients with RH poorly respond to it (10). Our study might help physicians identify patients with both FDD and RH timely in order to manage them more individually.

We detected RH in 50/311 (15.11%) and borderline RH in 37/331 (11.18%) of patients with FDD. More than a quarter of patients with FDD had one or more abnormally elevated sensory thresholds. This finding is consistent with an observational study where 163 of 667 constipated patients (24.4%) had one or more elevated thresholds (5). It is also suggested that there is a smaller proportion of constipated patients with ≥2 elevated sensory thresholds (13–17%) (23, 25). However, we did not find that patients with FDD suffered more RH than generalized constipated patients.

The underlying mechanism of how RH causes anorectal disorders is still unknown. The intact rectal sensation is fundamental to recto-anal and pelvic floor coordination (26). Some scholars hypothesize that individuals with RH have altered recto-anal reflexes and/or sensorimotor response, and the balloon volumes for inducing their rectoanal inhibitory reflex and contractile reflex were higher (27). Another study showed that patients with RH have reduced rectal wall contractility in response to distension, which likely contributes to failed defecation (28). However, we only found that the anal relaxation rate was lowest in the RH group but no difference in anorectal pressure or presence of pelvic floor disorder was observed among the three groups. As to comparisons of three rectal sensory thresholds, the presence of abnormally elevated FCSV, DDV, and MTV were all higher in patients with RH compared to those with borderline RH and non-RH, especially for DDV which was elevated in all patients with RH. We speculated that DDV might be a useful indicator for impaired rectal sensation. Only weak correlations were seen between FCSV and anal resting pressure, maximum squeeze pressure, and residual pressures, which is of limited clinical significance.

It is demonstrated that an increasing number of elevated sensory thresholds was associated with a more severe constipation phenotype (23). In our study, FDD patients with RH had more severe defecation symptoms. Meanwhile, the BSFS score was lower in these patients, indicating that they suffered from the lumpy or hard stool. The hard stool is closely related to RH in patients with FDD. Thus, more patients with RH needed manual maneuvers to help defecate. The conscious withdrawal of attention from rectal sensations or habitual suppression of the desire to defecate may contribute to impaired call to stool, which could cause rectal impaction and secondary dilatation of the rectum, leading to RH (29–31). The longer stool stays in the colon and rectum, the harder it may become, which could explain the lumpy or hard stool which patients with RH frequently have. Thus, these patients with FDD experience more severe defecation symptoms and need to use digital assistance or enema. Defecation symptom severity was correlated to QOL in these patients, which needs more attention.

We found that patients with RH were older and age was positively correlated to three rectal sensory thresholds, suggesting the decreased rectal sensation might be related to aging. Age-related impairment in the mechanoreceptors of the rectal wall and the pelvic afferent nerves might play a role in this relationship (32). A previous study by our team had a similar finding in a general functional constipation population (10). In addition, the proportion of male patients was high in the RH group. It is known that female patients are prone to constipation but most of them suffer slow transit constipation compared with male patients. A previous study found that constipated male patients were significantly more likely to suffer from defecation disorder than female patients (33). Additionally, our team has found that male patients tended to have much more paradoxical anal sphincter contraction and impaired anal sphincter relaxation (34). We speculated that male patients might have higher stress and various pressure than female patients and they are inclined to suppress the stool calling, which could lead to RH and FDD. Based on the analysis of a large patient cohort, older age and male sex were associated with higher rectal sensory thresholds (35), which agrees with our findings. However, the pathophysiological mechanism is still unknown and warranted to be explored in future studies.

The visceral sensation may be influenced by personality profile, autonomic nervous system function, and psychological phenotype (36, 37). However, little evidence has yet to be found directly in patients with RH. In our study, depression symptom was positively related to MTV, though the link is weak. But anxiety symptom was not related to any sensory threshold. The concept of the brain-gut axis is well recognized and peptide hormones (neuropeptide Y, peptide YY, glucagon-like peptide 1, etc.) released from the gut play a critical part in the interaction between the brain and digestive system (38, 39). Our results revealed that GAD-7 and PHQ-9 scores were positively related to defecation symptom severity. Patients with irritable bowel disease (IBS) (40) mostly have acute rectal feelings and psychological distress may aggravate IBS symptoms (41). It is established that anxiety can enhance visceral feelings (42, 43) but the effect of depression on sensation is controversial. According to our findings, we speculate that depression rather than anxiety plays an important role in blunt rectal sensation. However, logistic regression revealed that anxiety or depression was not related to the occurrence of RH. The association between mental state and RH in patients with FDD has been rarely studied and needs to be explored in future research.

We acknowledged that there are some limitations regarding our study. First, our study focused on patients with FDD in a single tertiary center, which unavoidably ended up with a highly selected population so the results could not be generalized to a wider primary care population. Second, volumetric balloon distension instead of barostat was used to test patients’ rectal sensory thresholds. Constipated patients with RH usually have persistent dilatation of the rectum and greater volumes will be required to stimulate the rectum (44). Thus, constipated patients with RH who have elevated volume thresholds might not have impaired rectal sensation actually. Recording pressure thresholds with barostat rather than volume thresholds is of more physiological significance (45). Nevertheless, in routine clinical practice, volumetric balloon distension is well accepted and often used (12, 46). In the future, testing rectal pressure thresholds with barostat may be a better and more rigorous method to identify RH. Finally, the link between constipation and RH is well established, but the cause–effect relationship in observational studies is still unclear. RH could lead to harder stool and more difficult defecation and long duration or severe constipation may result in a dilated rectum and abnormal rectal wall compliance which impairs rectal sensation and vice versa. Advanced prospective researches and cohort studies are in need.

This study has summarized the characteristics of FDD patients with RH by investigating symptomology, mental state, QOL, and functional tests. It is shown that patients with RH are older, more male patients, and vulnerable to suffering more severe defecation symptoms. Elevated DDV is most frequently seen in FDD patients with RH. Although abnormal motility and sensation may interact with each other and induce defecation disorder, we did not find specific links between them. Older age, gender (male), and lumpy or hard stool are related factors of RH in FDD and depression is associated with elevated MTV. The above findings may help physicians identify high-risk patients more efficiently. Thus, FDD patients with RH could get much better management in time.

## Data availability statement

The original contributions presented in the study are included in the article/supplementary material, further inquiries can be directed to the corresponding author.

## Ethics statement

Written informed consent was not obtained from the individual(s) for the publication of any potentially identifiable images or data included in this article.

## Author contributions

YJ and YT designed the study. YJ, YW, and MW collected and analyzed the data. YJ wrote the manuscript. YJ, YT, and LL revised the manuscript. All authors contributed to the article and approved the submitted version.

## Funding

This study is supported by National Natural Science Foundation of China (No. 81870378 and 82170556) for YT.

## Conflict of interest

The authors declare that the research was conducted in the absence of any commercial or financial relationships that could be construed as a potential conflict of interest.

## Publisher’s note

All claims expressed in this article are solely those of the authors and do not necessarily represent those of their affiliated organizations, or those of the publisher, the editors and the reviewers. Any product that may be evaluated in this article, or claim that may be made by its manufacturer, is not guaranteed or endorsed by the publisher.

## Abbreviations

RH: Rectal hyposensitivity
FDD: Functional defecation disorder
QOL: Quality of life
HR-ARM: High-resolution anorectal manometry
BET: Balloon expulsion test
MDI: Manometric defecation index
RAG: Recto-anal pressure gradient
FCSV: First constant sensation volume
DDV: Defecatory desire volume
MTV: Maximum tolerable volume
SBMs: Spontaneous bowel movements
BSFS: Bristol Stool Formation Scale
PAC-SYM: Patient assessment of constipation symptoms
GAD-7: General anxiety disorder 7-item
PHQ-9: Patient health questionnaire-9
PAC-QOL: Patient assessment of constipation quality of life
